# Hand hygiene practices for prevention of health care-associated infections associated with admitted infectious patients in the emergency department: a systematic review

**DOI:** 10.1007/s11845-022-03004-y

**Published:** 2022-04-18

**Authors:** M Issa, SS Dunne, CP Dunne

**Affiliations:** 1 School of Medicine, University of Limerick, Master’s in Public Health Programme, Limerick, Ireland; 2 Centre for Interventions in Infection, Inflammation & Immunity (4I) and School of Medicine, University of Limerick, Limerick, Ireland

**Keywords:** Emergency department, HAI, HCAI, Hand hygiene, Hand washing, Hand hygiene compliance, Health care-associated infection, Hospital-acquired infection, Systematic review

## Abstract

**Background:**

In most high-income countries, emergency departments (ED) represent the principal point of access forcer by critically ill or injured patients. Unlike inpatient units, ED healthcare workers (ED HCWs) have demonstrated relative lack of adherence to hand hygiene (HH) guidelines, commonly citing frequency of intervention and high rates of admission, which reflect severity of cases encountered.

**Aim:**

Assessment of studies on hand hygiene compliance (HHC) by ED HCWs conducted between 2010 and 2020, seeking to estimate HHC rates and intervention strategies utilised to improve HHC in EDs.

**Methods:**

Searches conducted in Web of Science, EBSCO HOST (CINHAL & Medline), PubMed, Embase, and Cochrane for full studies published between 2010 and 2020 on the topic of HHC in the ED.

**Results:**

One hundred twenty-nine eligible articles were identified of which 79 were excluded. Fifty-one underwent full-text screening before 20 studies were deemed relevant. Of the eligible studies, fifteen (75%) had, as the primary outcome, HHC according to the WHO-recommended 5-moments. Twelve studies (60%) implemented multimodal or single intervention strategies. Eight studies were ambiguous regarding the nature of the approach adopted. In the nine observational studies where HHC was documented, an overall post-intervention median HHC rate of 45% (range 8–89.7%).

**Conclusion:**

Multimodal approaches appear to have enhanced HHC moderately among ED HCWs. Elevated complexity associated with critically ill patients, and ED overcrowding, are contributing factors to relatively low compliance rates observed. Strategies to improve HHC rates may need to acknowledge, and cater for, the context of an unpredictable environment.

## Introduction

Health care-associated infections (HCAIs) are commonplace. The term encompasses a range of infections that may be contracted by a patient during a stay in the hospital for a pathology other than that infection [1]. Such healthcare-associated infections may, similarly, be contracted following contact with health services in clinics, long term care facilities, etc. Collectively, these infections represent a serious public health challenge linked with increased patient morbidity and mortality, and an economic burden for healthcare systems [2]. Unsurprisingly, infection prevention and control practices in developed countries have targeted reduction of HCAI impact, resulting in approximately 10% of hospitalised patients in higher-income countries affected. However, developing countries report considerably higher incidence (in some cases, greater than 25% of all hospitalised infections) [3]. In Europe, prevalence rates of HCAIs fluctuate between 4.6% and 9.3% [4].

The financial costs related to HCAIs are well understood, estimated at between $28 and $45 billion [5]. In the UK, public hospitals registered more than 650,000 HCAIs in 2016/17 amongst 13.8 million inpatients, including 22,800 fatal cases [6]. According to Stewart et al., the HCAI may create a further 7.8 days to the patient's hospital length of stay (LOS) with a median LOS of 30 days for HCAI patients and 3 days for non-HCAI patients. The paper concluded that a 10% decline in HCAI incidence could probably free up to 5800 bed-days [7]. It has been estimated that the NHS spent almost £2.1 billion on HCAIs in 2016/17 [6].

Causes of HCAIs are also reasonably well understood and relate mainly to patient and health care worker (HSW) transmission of potentially pathogenic microbes, often involving contamination of surrounding or nearby surfaces (e.g., equipment, clothing, sanitary ware) [8–10]. Efforts to mitigate risk of such transmission has focused, to a great extent, on hand hygiene (HH) [11], epitomised by the 2009 World Health Organisation “5 Moments for Hand Hygiene” [12]. In that context, Chen et al. estimated that US$25 can be saved for each US dollar spent on HH awareness [13].

Notably, HH compliance (HHC) in the emergency department (ED) has remained recalcitrant to significant improvement [14–17]. This is particularly challenging as, in most developed countries, the ED is the principal point of access for life-saving assistance by critically ill or injured patients [18]. Distinct from inpatient units, the ED environment and practices reflect elevated complexity associated with critically ill or polytrauma patients and overcrowding; contributing factors to the relatively low compliance rates observed. More specifically, Muller et al. [16] reported that ED crowding contributes to lower ED hand hygiene compliance highlighting typical restricted physical spaces within EDs, relatively close proximity of patients and staff, and unpredictable acuity level of patients as some of the more important HHC challenges in the ED. They further refer to ED staff often consulting more patients per shift than colleague sin other specialties, and the fact that such consults commonly occur in corridors and exposed spaces due to overcrowding and attempts to expedite patient care; often without ready access to alcohol-based hand rub [16]. Venkatesh et al. [19] reported complementary observations. Paradoxically, the high frequency of invasive procedures performed in the ED represent an opportunity for improvement in HH and reduction in HCAIs [16].

It is unfortunate then that systematic reviews and meta-analyses of HHC in EDs are scarce. Seo et al. [20] completed an appraisal of interventions to improve HHC in Eds [20] (from 1948 to 2018); reporting that 80% of eligible papers applied multimodal or dual interventions to improve HHC albeit that only half of the included studies observed a HHC rate above 50%. The review, however, utilised narrow inclusion criteria and included only studies reported in English and Korean. Cartel et al. [2] conducted a review of common infection control practices in the ED setting, between 2002 to 2012, (finding HHC rates ranging between 7.7% and 89.7%). Given such disparity, it is compelling to conduct a more inclusive systematic review that focuses on handwashing in the ED for the prevention of nosocomial infections associated with admitted infectious patients.

## Methods

### Study design

Registered with PROSPERO (https://www.crd.york.ac.uk/prospero) as CRD42021234488 [21], we conducted searches in the following databases: Web of Science, PubMed, CINHAL & Medline (EBSCO Host), Cochrane and Embase, on any article related to ED hand hygiene compliance published between 1st January 2010 and 31st December 2020. The following search terms were used alone or in combination: PubMed—(hand hygiene) OR (handwashing) AND (emergency room OR emergency department OR ER OR ED) [Title / Abstract] ) AND compliance [Title / Abstract]; CINHAL and MEDLINE and EMBASE and Cochrane—(hand hygiene) OR (handwashing) AND compliance AND (emergency room OR emergency department OR ER OR ED); Web of Science—(hand hygiene) OR (handwashing) AND compliance AND (emergency room OR emergency department OR ER OR ED) AND article, a summary of published article [type of document].

Articles were deemed eligible if they described any intervention, peer-reviewed or grey literature, seeking to affect HHC by ED staff. In summary, this included observational studies, randomised and non-randomised controlled studies, before and after studies, and cross-sectional studies that investigated effectiveness of interventions on HHC in EDs determined either by observation or the use of the camera surveillance, with no limitation to English language or geographical location. Unlike previous reviews on the topic, our criteria encompassed all healthcare providers working in emergency departments: physicians, residents, nurses including assistant nurses and student nurses, healthcare assistants, technicians, physiotherapists, and any other ED personnel.

Specifically excluded were studies conducted outside the ED settings (ICU, prehospital setting, any ward, military bases, prisons, schools, etc.), editorial opinions, posters presentations (not full article), unpublished conference proceedings (not full article), letters to the Editor, opinion-based publications, doctoral theses, and quality improvement projects.

### Data extraction

Two reviewers (MI and CPD) hand-reviewed search results independently to identify articles meeting the inclusion criteria. Wherever inconsistencies arose, disagreement was discussed and resolved by consensus. Data extraction was completed by the reviewers separately and disagreement again resolved by consensus. A data extraction form was utilised that captured: the study design; year of publication; database source; country of the publication including World Bank regions and income classification; sample size; types and characteristics of the participants; recorded number of HH opportunities; HHC rate; study intervention; details of the intervention; strengths and weaknesses of the study; and outcomes. Meta-analysis could not be conducted due to abundant heterogeneity of the studies. Therefore, analysis was only possible by compiling results manually in tables.

### Assessment of bias risk in eligible studies

The reviewers (MI and CPD) analysed the methodological quality of the included studies. For observational studies, the Risk of Bias Assessment for Nonrandomized Studies (RoBANS) tool [22] was utilised. For cross-sectional study designs, the Appraisal tool for Cross-Sectional Studies (AXIS) was used to assess five elements of each study (Introduction, Methods, Results, Discussion and other) by answering 20 stipulated questions [23].

## Results

**Fig. 1:**
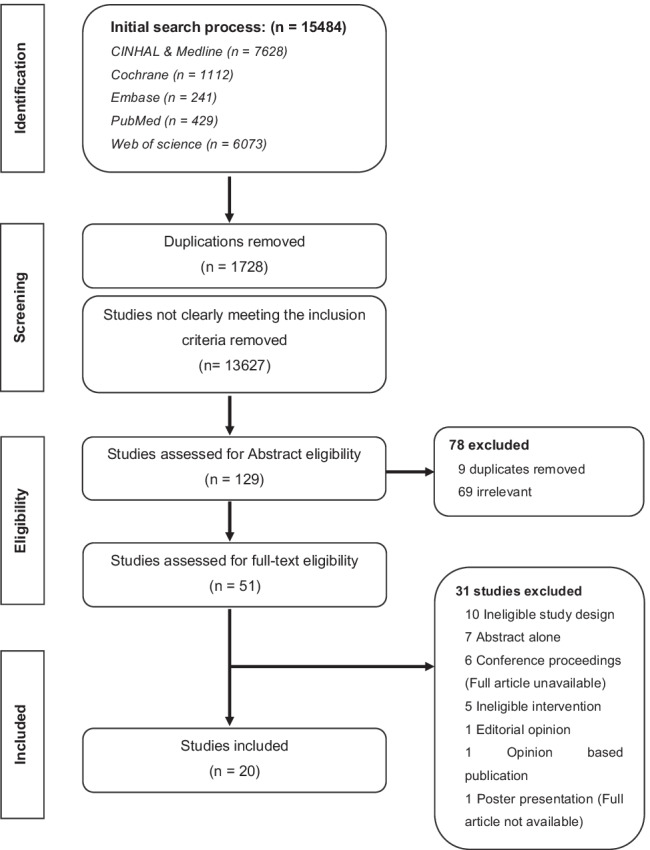
PRISMA flow diagram for the selection of studies

Following the search, 15,484 publications were retrieved of which 15,355 papers were excluded as duplicates or evidently ineligible (Fig. 1). Of the remaining 129 papers, a further 78 were excluded subsequent to abstract screening (60%), as conducted outside ED settings (69 papers, 53%) and additional duplicates were found (9 papers, 7%). Therefore, full-text appraisal was performed for 51 studies. Of those, 31 studies failed to fully meet the inclusion criteria, with 20 studies (16%) of HHC eventually included in this review (Table 1).

**Table 1:** Included articles

**Authors**	**Year**	**Title**	**Database**	**Language**	**Country**	**World Bank regions**	**World Bank income classification ** [24]
Arntz et al. [25]	2016	*Effectiveness of a multimodal hand hygiene improvement strategy in the emergency department*	Embase	English	Netherlands	Europe and Central Asia	High income
Carter et al. [26]	2016	*Environmental factors and their association with emergency department hand hygiene compliance: An observational study*	Web of Science	English	USA	North America	High income
di Martino et al. [27]	2011	*Assessing the sustainability of hand hygiene adherence prior to patient contact in the emergency department: A 1-year postintervention evaluation*	PubMed	English	Italy	Europe and Central Asia	High income
Espinoza Diaz et al. [28]	2018	*The microbial load and hand washing of the emergency department staff in the “hospital de especialidades josé carrasco arteaga”*	Cochrane	Spanish	Ecuador	Latin America & the Caribbean	Upper-middle income
Fouad et al. [29]	2020	*Hand hygiene initiative: Comparative study of pre- and postintervention outcomes*	CINHAL & Medline	English	Saudi Arabia	Middle East and North Africa	High income
Ghazali et al. [30]	2018	*Impact of a simulation-based training in hand hygiene with alcohol-based hand rub in emergency departments*	CINHAL & Medline	English	France	Europe and Central Asia	High income
Haac et al. [31]	2017	*Hand hygiene compliance in the setting of trauma resuscitation*	Embase	English	USA	North America	High income
Hong et al. [32]	2012	*Bacterial contamination of computer and hand hygiene compliance in the emergency department*	Web of Science	English	Republic of Korea	East Asia and Pacific	High income
Mahfoozpour et al. [33]	2013	*Minding the prevention protocol for blood-borne diseases *via* EM residents*	Embase	English	Iran	Middle East and North Africa	Upper-middle income
Martel et al. [34]	2013	*Respiratory hygiene in emergency departments: Compliance, beliefs, and perceptions*	Web of Science	English	Canada	North America	High income
Muller et al. [16]	2015	*Hand hygiene compliance in an Emergency Department: The effect of crowding*	PubMed	English	Canada	North America	High income
Parmeggiani et al. [35]	2010	*Healthcare workers and health care-associated infections: Knowledge, attitudes, and behavior in emergency departments in Italy*	Embase	English	Italy	Europe and Central Asia	High income
Reardon et al. [36]	2012	*Time burden of emergency department hand hygiene with glove use*	Embase	English	USA	North America	High income
Sakihama et al. [37]	2015	*Improving healthcare worker hand hygiene adherence before patient contact: A multimodal intervention of hand hygiene practice in three Japanese tertiary care centers*	Embase	English	Japan	East Asia and Pacific	High income
Scheithauer et al. [38]	2013	*Improving hand hygiene compliance in the emergency department: Getting to the point*	Web of Science	English	Germany	Europe and Central Asia	High income
Schmitz et al. [39]	2014	*Effectiveness of a multimodal hand hygiene campaign and obstacles to success in Addis Ababa, Ethiopia*	PubMed	English	Ethiopia	Sub-Saharan Africa	Low income
Stackelroth et al. [40]	2015	*Hesitation and error: Does product placement in an emergency department influence hand hygiene performance?*	CINHAL & Medline	English	Australia	East Asia and Pacific	High income
Strauch et al. [41]	2020	*Use of an automated hand hygiene compliance system by emergency room nurses and technicians is associated with decreased employee absenteeism*	CINHAL & Medline	English	USA	North America	High income
Venkatesh et al. [19]	2011	*Predictors of hand hygiene in the emergency department*	CINHAL & Medline	English	USA	North America	High income
Zottele et al. [15]	2017	*Hand hygiene compliance of healthcare professionals in an emergency department*	Embase	Portuguese	Brazil	Latin America & the Caribbean	Upper-middle income

### Details of the included studies

Of the 20 included articles (Table 1), four papers were cross-sectional studies, two studies were retrospective, and 14 publications were observational studies (Table 2). Notably, 18 papers (90%) were in English, one study (5%) in Portuguese, and one publication (5%) in Spanish (Table 1). Six of the papers (30%) were retrieved from CINAHL & Medline, six (30%) from Embase, three (15%) from PubMed, four (20%) from Web of Science, and one (5%) from Cochrane (Table 1).

**Table 2:** Study design, setting, and location of included articles

**Authors**	**Year**	**Study design**	**Study settings**	**Study duration**	**Country**
Arntz et al. [25]	2016	Before-and-after observational study	Single centre	May-Sep 2013	Netherlands
Carter et al. [26]	2016	Before-and-after observational study	Single centre	Nov 2017 – Mar 2018	France
di Martino et al. [27]	2011	Before-and-after observational study	Multicentre	(A) May-Jul 2013	Japan
				(B) Oct 2012	
				(C) Jun 2013	
Espinoza Diaz et al. [28]	2018	Before-and-after Observational study	Single centre	May—Aug 2012	Ethiopia
Fouad et al. [29]	2020	Cross-sectional study	Single centre	Sep 2017- Feb 2018	Ecuador
Ghazali et al. [30]	2018	Cross-sectional study	Single centre	Mar-May 2010	Iran
Haac et al. [31]	2017	Cross-sectional study	Multicentre	Part 1: 11–18 Feb 2010	Canada
				Part 2: 18–19 Feb 2010	
Hong et al. [32]	2012	Cross-sectional study	Multicentre	Apr 2006—Jun 2007	Italy
Mahfoozpour et al. [33]	2013	Observational study	Single centre	Oct 2013- Jan 2014	USA
Martel et al. [34]	2013	Observational study	Single centre	Mar-Jun 2009	Italy
Muller et al. [16]	2015	Observational study	Single centre	Oct 2016—Mar 2017	Saudi Arabia
Parmeggiani et al. [35]	2010	Observational study	Multi-centre	No data	Republic of Korea
Reardon et al. [36]	2012	Observational study	Single centre	Jan 2011 – Oct 2013	Canada
Sakihama et al. [37]	2015	Observational study	Single centre	-	USA
Scheithauer et al. [38]	2013	Observational study	Single centre	-	Australia
Schmitz et al. [39]	2014	Observational study	Single centre	Jan 2019- Apr 2020	USA
Stackelroth et al. [40]	2015	Observational study	Single centre	Mar -Jul 2015	Brazil
Strauch et al. [41]	2020	Retrospective observational study	Single centre	Aug -Sep 2015	USA
Venkatesh et al. [19]	2011	Retrospective observational study	Single centre	Jan 2015-July 2016 Aug 2016-Dec 2018	USA
Zottele et al. [15]	2017	Tri-phase observational study	Single centre	Feb-Sep 2011	Germany

### Geographic location

Using World Bank country classification [24], seven studies were found to have been conducted in North America (USA *n* = 5, Canada *n* = 2). Three were performed in East Asia & Pacific (Australia *n* = 1, Japan *n* = 1, Republic of Korea *n* = 1), five articles originated from Europe (Italy *n* = 2, France *n* = 1, Germany *n* = 1, Netherlands *n* = 1). Two studies originated in Latin America & the Caribbean (Brazil *n* = 1, Ecuador *n* = 1). The Middle East & North Africa generated two papers (Saudi Arabia *n* = 1, Iran *n* = 1) and one study was conducted in Ethiopia (Table 2).

Four cross-sectional studies (20%) were included in the review, each emanating from a different region (Canada, Ecuador, Iran, and Italy). Among the sixteen observational articles, four studies (20%) were before-and-after designs, conducted in Ethiopia, Netherlands, France, and Japan. Nine studies (45%) were descriptive observational papers from Australia, Brazil, Canada, Italy, Saudi Arabia, Korea, and the USA. Two of the twenty articles (10%) were retrospective observational studies performed in the USA, and one article (5%) described tri-phase observational research conducted in Germany (Table 2).

### Quality assessment results

Using the RoBANS tool [22] (Table 3), 14 studies had low risk of bias for selective outcome reporting, 16 had low risk of bias for incomplete outcome data, one paper had low risk of bias of blinding for outcome assessment, eight had low risk of bias for intervention (exposure) measurement, 11 had low risk of bias for confounding variables. All papers had high risk of bias for selection of participants. Four cross-sectional study designs were appraised using the AXIS tool (Table 4); all four associated with low risk of bias, albeit that two did not discuss the limitations of their studies.

**Table 3:** Risk of bias assessment tool for nonrandomized studies (RoBANS) [22]

**Authors**	**Year**	**Selective outcome reporting**	**Incomplete outcome data**	**Blinding of outcome assessment**	**Intervention (exposure) measurement**	**Confounding variables**	**Selection of participants**
Arntz et al. [25]	2016	Low	Low	Unclear	Low	Low	High
Carter et al. [26]	2016	Low	Unclear	High	High	Low	High
di Martino et al. [27]	2011	Low	Low	High	High	Low	High
Fouad et al. [29]	2020	Low	Low	High	Low	Low	High
Ghazali et al. [30]	2018	Low	Low	High	Low	Low	High
Haac et al. [31]	2017	Low	Unclear	Low	Low	Unclear	High
Hong et al. [32]	2012	Low	Low	High	Low	Low	High
Muller et al. [16]	2015	High	Low	High	High	Unclear	High
Reardon et al. [36]	2012	Low	Low	High	High	Low	High
Sakihama et al. [37]	2015	Low	Low	High	High	High	High
Scheithauer et al. [38]	2013	Low	Low	High	Low	Low	High
Schmitz et al. [39]	2014	Low	Low	Unclear	Low	Low	High
Stackelroth et al. [40]	2015	Low	Low	Unclear	High	High	High
Strauch et al. [41]	2020	Low	Low	High	High	High	High
Venkatesh et al. [19]	2011	Low	Low	High	Low	Low	High
Zottele et al. [15]	2017	High	Low	High	High	Low	High

**Table 4:** The appraisal tool for cross-sectional studies (AXIS tool): Yes, No, Don’t know [23]

**Major Components**	Espinoza Diaz et al. [28]	Mahfoozpour et al. [33]	Martel et al. [34]	Parmeggiani et al. [35]
**Introduction**				
1. Were the aims/objectives of the study clear?	Yes	Yes	Yes	Yes
Methods				
2. Was the study design appropriate for the stated aim(s)?	Yes	Yes	Yes	Yes
3. Was the sample size justified?	Yes	Yes	Yes	Yes
4. Was the target/reference population clearly defined? (Is it clear who the research was about?)	Yes	Yes	Yes	Yes
5. Was the sample frame taken from an appropriate population base so that it closely represented the target/reference population under investigation?	Yes	Yes	Yes	Yes
6. Was the selection process likely to select subjects/participants that were representative of the target/reference population under investigation?	Yes	Yes	Yes	Yes
7. Were measures undertaken to address and categorise non-responders?	Don’t know	Yes	Yes	Yes
8. Were the risk factor and outcome variables measured appropriate to the aims of the study?	Yes	Yes	Yes	Yes
9. Were the risk factor and outcome variables measured correctly using instruments/ measurements that had been trialled, piloted, or published previously?	Yes	No	Yes	Yes
10. Is it clear what was used determined statistical significance and/or precision estimates? (e.g., p values, CIs)	Yes	Yes	Yes	Yes
11. Were the methods (including statistical methods) sufficiently described to enable them to be repeated?	Yes	Yes	Yes	Yes
**Results**				
12. Were the basic data adequately described?	Yes	Yes	Yes	Yes
13. Does the response rate raise concerns about non-response bias?	No	No	No	No
14. If appropriate, was information about non-responders described?	No	No	No	No
15. Were the results internally consistent?	Yes	Yes	Yes	Yes
16. Were the results for the analyses described in the methods, presented?	Yes	Yes	Yes	Yes
**Discussion**				
17. Were the authors' discussions and conclusions justified by the results?	Yes	Yes	Yes	Yes
18. Were the limitations of the study discussed?	No	No	Yes	Yes
**Others**				
19. Were there any funding sources or conflicts of interest that may affect the authors’ interpretation of the results?	No	No	Yes	Yes
20. Was ethical approval or consent of participants attained?	Yes	Yes	Yes	Yes
**Overall assessment / Risk of bias**	**Low**	**Low**	**Low**	**Low**

### Study objectives

Of the 20 articles, 55% (*N* = 11) intended to evaluate HHC among ED HCWs based on the WHO-recommended 5-moments of hand hygiene. Fifteen percent (*N* = 3) assessed the effect of ED crowding on ability of HCWs to correctly perform HH. Similarly, fifteen percent (*N* = 3) estimated the bacterial loads of HCWs hands (*N* = 2) or equipment (*N* = 1) within EDs., while the same number (*N* = 3) assessed compliance using ABHR solutions (Table 5).

**Table 5:** Study objectives of included articles

**Authors**	**Year**	**Study Objectives**
Arntz et al. [25]	2016	To assess the effect of a multimodal improvement strategy on HHC in the ED
Carter et al. [26]	2016	To assess the sustainability of the hand hygiene intervention in the paediatric ED
di Martino et al. [27]	2011	The primary aim was to assess the duration of hand rubbing before and after a simulation-based training course Secondary objectives: (1) to count the number of steps carried out, (2) to analyse the quality of HH and (3) to note the presence of jewellery
Espinoza Diaz et al. [28]	2018	To quantify opportunities for HH according to WHO guidelines, and to measure HHC and glove use during active resuscitation of trauma patients in the Trauma Resuscitation Unit (TRU)
Fouad et al. [29]	2020	To determine the compliance with respiratory hygiene of triage nurses at 2 university hospitals and to identify factors influencing compliance with the respiratory hygiene principles of emergency HCWs
Ghazali et al. [30]	2018	This survey assessed knowledge, attitudes, and compliance regarding standard precautions about HAIs and the associated determinants among HCWs in ED in Italy
Haac et al. [31]	2017	To assess a multimodal HH intervention coupled with a contest to improve HHC
Hong et al. [32]	2012	To assess the impact of implementing a WHO-recommend multimodal HH campaign at an Ethiopian hospital
Mahfoozpour et al. [33]	2013	To improve the hand hygiene practices of emergency room nurses and technicians
Martel et al. [34]	2013	To analyse compliance with hand hygiene by healthcare professionals in an emergency department unit
Muller et al. [16]	2015	To assess the rate of adherence to preventive measures (PM) against blood-borne diseases via emergency medicine residents
Parmeggiani et al. [35]	2010	To examine the relationship between crowding and HHC in the ED
Reardon et al. [36]	2012	To evaluate whether ED crowding is also associated with reduced HHC among HCWs
Sakihama et al. [37]	2015	To describe the prevalence of HHC in a large urban ED and identify the predictive value of HCW type, ED layout, and workflow on HH in the ED
Scheithauer et al. [38]	2013	To study the behaviour of HH and determine the microbial load on the hands of HCWs in the ED
Schmitz et al. [39]	2014	To determine the effectiveness of infection control intervention to improve compliance with hand hygiene in the Emergency Department To evaluate bacterial load on hands as a possible indicator of improvement
Stackelroth et al. [40]	2015	To determine the degree and nature of bacterial contamination of multiple user computer equipment (computer keyboards and electronic mice) located in three EDs at urban teaching hospitals in Korea
Strauch et al. [42]	2020	To assess the use of ABH cleanser in the ED with non-sterile glove use
Venkatesh et al. [19]	2011	(1) To define the number of hand-rubs needed for individual patient care at the ED (2) To optimize HHC without increasing workload
Zottele et al. [15]	2017	To examine the extent to which incorrect hand hygiene occurred because of the inability to easily distinguish between different hand hygiene solutions placed at washbasins

### Study design and settings

Of the included studies, only four (20%) were multicentre studies, with the remainder performed in discrete EDs in single hospitals (Table 2).

### Healthcare worker category and time and place of intervention

Although all studies stipulated HCW categories involved, 12 of 20 studies (60%) did not provide specific detail of included participants differentiated by profession. Irrespective, HCWs involved were mostly nurses and nurse's assistants, physicians of all grades, healthcare assistants, technicians, and ‘other’ staff (Table 6). More specifically, 16 of the 20 articles listed nurses, 13 studies associated physicians, seven papers included residents, seven studies cited technicians, and six studies involved nursing assistants among their population sample.

**Table 6:** Health care workers category and time/place of interventions

**Authors**	**Year**	**Study population**	**Time/ place of intervention**
Arntz et al. [25]	2016	Nurses (*n* = 39) Physicians (*n* = 14) Staff physicians (*n* = 5)	-Baseline Observation: Day, evening (week and weekends) -Intervention observation: only weekdays and evening
Carter et al. [26]	2016	Nurses (*n* = no data) Physicians (*n* = no data) Nursing assistants (*n* = no data) Others (therapists, radiology, technicians, security…) (*n* = no data)	Day vs night shifts Patient location: hallways, Semi-private, private NEDOCS crowding score: - < 100: ED not crowded -101–140: Ed overcrowded -141–180: Ed severely overcrowded -181 + : ED dangerously overcrowded
di Martino et al. [27]	2011	Nurses (*n* = no data) Physicians (*n* = no data) Staff physicians (*n* = no data)	1 year after the intervention 16 sessions of 28.5 observations per session, average 33 min per session (20–50)
Espinoza Diaz et al. [28]	2018	Nurses (*n* = 10) Nurse assistants (*n* = 10) Physicians (*n* = 10) Residents (*n* = 10) Interns (*n* = 20)	Not stated
Fouad et al. [29]	2020	Nurses & nurse students (*n* = no data) Physicians & students (*n* = no data) Other: therapists/ dietitians/ technicians/ dentists / students (*n* = no data)	Randomly distributed throughout the day and night
Ghazali et al. [30]	2018	Nurses (*n* = 12) Nursing assistants (*n* = 4) Residents (*n* = 6)	During workdays
Haac et al. [31]	2017	Nurses (*n* = no data) Physicians (*n* = no data) Technicians (*n* = 29) Students (*n* = 39)	Weekdays, weekends and day and night shifts
Hong et al. [32]	2012	Nurses (*n* = no data) Physicians (*n* = no data)	24 h in each of the 3 EDs
Mahfoozpour et al. [33]	2013	80 EM residents	
Martel et al. [34]	2013	Nurses (*n* = no data) Physicians (*n* = no data) Residents (*n* = no data) Medical students (*n* = no data) Other (*n* = no data)	Day, evening, night shifts
Muller et al. [16]	2015	Nurses (*n* = no data) Physicians (*n* = no data) Other-nursing assistant, housekeeping, transport, IV team, radiology technicians, nursing students (*n* = no data)	Monday to Friday in all ED areas
Parmeggiani et al. [35]	2010	550 ED staff from 8 hospitals included	
Reardon et al. [36]	2012	Nurses (*n* = 20) Physicians (*n* = 2) Residents (*n* = 11) Physician assistants (*n* = 4) Medical students (*n* = 3)	Day and night shifts in adult ED
Sakihama et al. [37]	2015	Nurses (*n* = no data) Physicians (*n* = no data)	Monday to Friday 08:30–19:30
Scheithauer et al. [38]	2013	Nurses (*n* = no data) Physicians (*n* = no data) Medical students (*n* = no data) Trainees (*n* = no data)	Not stated
Schmitz et al. [39]	2014	Nurses (*n* = no data) Physicians (*n* = no data) Resident physicians (*n* = no data) Medical students (*n* = no data) Other (*n* = no data)	Day shifts All hospital wards, except Paeds ward
Stackelroth et al. [40]	2015	All ED staff and visiting non-ED like ambulance officers (no data)	Day 10: 24 h observation
Strauch et al. [41]	2020	Nurses (*n* = no data) Technicians (*n* = no data)	Not stated
Venkatesh et al. [19]	2011	Nurses (*n* = no data) Physicians (*n* = no data) Physician assistants (*n* = no data) Nurse assistants (*n* = no data) Transport (*n* = no data) Other (*n* = no data)	All shifts and all days of the week between 7 a.m. to 2 a.m
Zottele et al. [15]	2017	Nurses (*n* = 17) Resident physicians (*n* = 12) Nurse technicians (*n* = 28) Physiotherapists (*n* = 2)	Morning, afternoon, and night shifts

With respect to timing and location at which observations were conducted, most studies ran across days, evening and nights shifts throughout both weekdays and weekends. Exceptions were Muller et al. [16] who did not include weekends shifts in their investigation, and Sakihama et al. [37] and Schmitz et al. [39] who conducted their study only during day shifts. Notably, some studies focused on the influence of location within EDS on HHC, including Cartel et al. [26] who included in their observation the location of patients such as hallways, semiprivate and private ED areas to estimate the effect of crowding on HHC. Use of technological surveillance was detailed by Strauch et al. [41] who used a retrospective reading of an electronic device to track the HH activity data on each personalised badge when returned to a charging station.

### Study interventions

Twelve studies (60%) implemented a specific strategy to enhance HHC within the ED either by multimodal or single intervention strategy. Education and training were employed in 9 of the 20 studies (45%) [25, 27, 29–31, 34, 37–39]. Reminders applied in 4 of the 20 articles (20%) [25, 37–39]. The use of feedbacks was highlighted in three publications (15%) [25, 37, 38]. Emails to alert participants were sent by three authors (15%) [26, 40, 41]. Posters were utilised by two authors (10%) [25, 38]. Evaluation and Questionnaires were reported in three articles (15%) [30, 34, 37]. Feedback approaches were associated with three publications (15%) [25, 37, 38]. Two authors incorporated the method of role models in their study (10%) [27, 39]. Video training was associated with Ghazali et al. [30], while two authors included presentations to improve the HCWs HH practice [30, 38]. Simulation and live demonstration were incorporated in Fouad et al.'s article [29]. Eight studies (40%) were unclear in terms of their intervention. Amongst them, seven papers (35%) used direct observations techniques [15, 16, 19, 28, 32, 33, 36] and one study employing a video surveillance approach [31] (Table 7).

**Table 7:** Study interventions and compliance outcome

**Authors**	**Year**	**improvement strategies**	**Intervention**	**Detail of intervention**	**Measurement’s methods**	**Main outcome**
Arntz et al. [25]	2016	Infrastructure improved No changes in HH sinks	-Education -Reminders -Computer screensavers of 5HH -Feedbacks	-Observations immediately followed: Baseline week & 3 interventions weeks Undercover nurses appointed to provoke participants (intervention 3)	-2 medical students blinded to participants -Received training on 5HH -Compliance measured according to WHO 5HH	HHC HHC rate
Carter et al. [26]	2016		HCW informed via email and shifts huddles regarding the planned study	-60 min HHC direct observation -HH techniques not assessed -Maximum of 3 HH opportunities per HCW per observation period	-4 trained research associates observed HHC according to WHO 5HH -Marked only if HH was performed -HCW types, Glove use, Nursing staffing levels, Day of the week, Shift of observation, HH indication & Location of a patient within the ED	HHC
di Martino et al. [27]	2011	All HCWs were informed about the goals of the observations	HHC assessed in 3 phases: baseline-intervention-post intervention -HCWs received formal training -Role model promoting HH and wearing a reminder button -ABHR provided -Creation of a sense of urgency	-All HCWs were informed about the goals of the observations -Baseline data of HHC rate presented to HCWs	-2 observers made observations in all 3-time points (Preintervention, immediately post-intervention & 1-year post-intervention)	HHC 1 year post intervention
Espinoza Diaz et al. [28]	2018			-HH practices followed by a collection of microbiological samples from HCWs washed hands -Interviews of HCWs on the clinical HH techniques followed by the microbiological assessment of hands after HH	-Samples sent to the laboratory within 24 h -The microbiological count was done by an approved laboratory	HHC Microbial load on the hands of HCWs
Fouad et al. [29]	2020		HH education programme offered to ED staffs: -Live demonstration, -Posters of 5HH placed in strategic areas in ED -Availability of ABHR and PPE	HHC assessed in 3 phases: -Phase I: (Baseline): HHC measured by direct observation -Phase II (intervention phase) -Phase III-Post intervention: HHC level measured to determine the effect of the intervention HAI rate assessed by determining hand bacterial load	-Trained volunteer students conducted observations -HCWs hands inserted into a sterile polyethylene bag of 50 ml of sterile peptone. Hands massaged for 1 min	WHO 5HH
Ghazali et al. [30]	2018		-Presentation -Monthly reminders -Training videos on HH -Simulation-based training (SBT) -Self-assessment and questionnaire	-Use of a timer to assess hand-rubbing duration -Hand-rubbing videotaped to count the number of steps	-2 trained independent observers performed an anatomical based assessment scale for HH with ABHR with a binary score: (0) not cleaned and (1) cleaned zones -Analysed 22 areas of the dorsal side and 18 areas of the palmar side of each hand -Assessor followed participants during workdays -Use of blue fluorescence to attest to the efficiency of ABHR	Duration of hand rubbing before and after a simulation course
Haac et al. [31]	2017		HCWs received training on 5HH during orientation and annual competencies	-Video surveillance of the TRU with 3 views -Anonymous observers to audit HHC on bay entry/exit -Surgical resident extracting data according to prespecified training	-HCWs unaware that camera was used for HHC monitoring -HCW-patient interaction from the moment HCW enters the patient bay to the final exit -Videotape reviewed as many times as possible to assess HHC	WHO 5HH
Hong et al. [32]	2012			-Staff HHC observed for 20 min period every 4 h for 24 h -Observer introduced himself to participants -Then maintain a discreet presence to avoid interference -All observations recorded manually	-Microbiological sample from all computers in all 3 EDs obtained -Keyboards sampled by moving sterile swab over all keys over 60 s -Each mouse was sampled at palm rest, left, and right-click and standard 6cm^2^ areas -Specimens incubated at 35 °C for 48 h daily inspection of colonies by a clinical microbiologist	WHO 5HH
Mahfoozpour et al. [33]	2013			The form had 2 sections: -Sect. 1 with 13 items -Sect. 2 with 10 items For each sample, 4 observations were performed by 1 observer from which the last 3 were considered as part of the research	-2 ID specialists and 1 epidemiologist designed an observation form for data collection. Using the data collection form, EMR minding PM against BDD was rated as Poor (not done, score 0), Fair (incompletely done, score 1) and Good (completely done, score 2) -A second form assessed different excuses for not minding the PM against BDD	The adherence rate to PM against BDD
Martel et al. [34]	2013		PRECEDE approach: Predisposing, reinforcing, and enabling factors in Educational Diagnosis and Evaluation	**Part 1:** 5 medical students observed ED nurses in the presence of a patient with fever and cough **Part 2:** A questionnaire was distributed to HCWs voluntarily with a Likert scale from 0–4	-Anonymous observation of nurses in triage -Shifts observed, sex of HCWs -A form with 9 items was designed for compliance rate, which included HH after and before the patient contact -A questionnaire used in part 2 to assess baseline characteristics of participants was distributed	-Respiratory hygiene compliance -knowledge of respiratory hygiene by ED staff
Muller et al. [16]	2015			-Patient volumes, staffing levels, time to physician assessment, and time to patient discharge were used as markers of ED crowding	-A trained observer randomly selected a specific ED room/bay and observed staff for 20 min -HH indications, professional group, day of the week and time of the day shift also measured	-WHO 5HH (Summarised moments 4&5 in one single moment 4)
Parmeggiani et al. [35]	2010		Small pilot test of questionnaire on knowledge about HCWs and control measures with 30 volunteer HCWs	-Five questions asked: demographic & occupation characteristics, knowledge about the risk of HAIs to patients, attitudes towards precautionary guidelines and perception of risk, the practise of standard precautions and source of up-to-date information		
Reardon et al. [36]	2012			-Participants blinded to study objective -Participants asked to put on gloves as they would before placing the nasogastric tube -Participants donned gloves once with hand rub and once without hand rub, serving as their own control	-Record of indication of glove use based on WHO 5HH -Record of any missing indication by the physician -Calculation of frequency of gloving episodes per hour, number of patients seen per hour and number of patient interactions per hour -The proportion of hand rubbing episodes lasting 20 s or more	To assess the use of ABH cleanser in the ED with non-sterile glove use
Sakihama et al. [37]	2015	Hospitals (A) & (B) hired an infection control nurse before intervention Hospital (C) had a dedicated infection prevention department Each facility received training on HHC prior	Multimodal HH intervention: -Contest of 5000USD -Infrastructure -Training/education -Evaluation, Feedback -reminder and institution safety climate	-Contest with a 5000USD reward for the outperforming hospital post-intervention	-HH adherence rate was tracked before patient contact for each unit and hospital and compared these to pre-intervention adherence rates -A qualified infection control nurse conducted all HHC observations	WHO 5HH
Scheithauer et al. [38]	2013		-Individual teachings -Direct feedbacks -Use of SOPs optimised for invasive procedures during phase I&II to improve workflow practices and HHC -Compilation of flowcharts -Presentation of interim results as a training and motivational tool	-Three 6-week observation phases interrupted by two 6-week interventions -Started after a 6-week pilot phase for familiarization, adapt WHO recommendations and minimize the effects because of observation	-Anonymous observation by a qualified infection control nurse -WHO 5HH recommendation -HR recorded from the use of sterile and nonsterile gloves -Glove usage instead of HR was documented	WHO 5HH
Schmitz et al. [39]	2014	-Soap and waterless hand sanitiser available -Refills were always available -Soap made available at all sinks	-Training & Education in English and Amharic to all HCWs -Ongoing postintervention training sessions -Visual reminders for HH -Development of institutional safety climate (HH champions or role models or HH facilitators)	-Baseline intervention over 4 weeks included a series of direct observations -The intervention phase over 6 weeks included the implementation of an abbreviated version of the WHO 5HH -Post-intervention phase over 4 weeks, immediately after the intervention phase	-Single observer trained on WHO 5HH performed all HH observation -HHO were adapted from the original WHO 5HH -Date, location, profession category, indication, and if the encounter occurred on attending physician rounds were all recorded	
Stackelroth et al. [40]	2015	Camera installed to observe HH practices	2 weeks prior start, information sheet was emailed to ED staff	-Participants aware of video surveillance but unsure of specific variables being measured -Direct observational method was used using ceiling-mounted, motion activated video camera surveillance on 24-h data of Day 10 out of 18 days used for recording	HHC based on Larson & Lusk’s modified Friedman’s criteria [42] : -What product used by individual to clean hands? -Does the individual use an incorrect product for HW? -Does the individual display hesitation during the selection of HH product? Measurement hesitation	HH practices
Strauch et al. [41]	2020	The use of automated HHCS	-Wearable badge detecting HHC before and after patient room entry by using different reminders via lights and tones -Staff received weekly emails of their performance -Manager and infection control received weekly/monthly data for coaching, performance tracking	-**Green light:** HH performed as required -**Yellow light:** reminder to clean hands -**Red light:** HH not performed within required time (non-compliant)	-HH activity data collected on each badge and uploaded to cloud based system when badge returned to a charging station -Preintervention: HH monitored through direct observation by an unknown observer -Daily sick calls of nurses and technicians collected from ER records -Total sick call hours for each day averaged within each month	Reduction of number of sick calls for nurses and technicians
Venkatesh et al. [19]	2011			-HHC for each HHO while checking ED units, bed location, room visibility -ED workflow included glove use, before and after patient contract, weekday’s vs weekends, and time of the day	-5 trained observers and 1 infection control specialist conducted HHC observations for 30–90 min observation periods -HCWs were aware of the observer’s role	WHO 5HH either by ABHR or standard handwashing
Zottele et al. [15]	2017		Seven sessions of pilot observations carried out in the ED to introduce the observer, increase familiarity, and reduce Hawthorne effect	-Single trained observer conducted the observation in room 1 (clinically stable patients) of approximately 20 min during the 3 work shifts -Excluded observation during surgical emergencies	-Single trained observer conducted the data collection using a worksheet with the schedule of the participants -HHC was calculated by dividing the number of HH actions performed by HCWs and the number of HHO, multiplied by 100	WHO 5HH

Arntz et al. [25] and Schmitz et al. [39] utilised additional education, feedbacks, and reminders, while Sakihama et al. [37] recruited a new infection control specialist in 2 of their 3 centres to assist with improvements. Di Martino et al. [27] provided formal training and employed role models wearing reminder buttons to promote HH. Fouad et al. [29] mounted live demonstrations and posters as nudges towards change. Of interest, Stackelroth et al. [40] installed a video surveillance system but without clear HH training for staff. In contrast, Sakihama et al. [37] supplemented available training and then instigated a competition to reward best performing groups while, in a questionable aspect to their study, Zottele et al. [15] organised seven sessions with their study observers to increase their familiarity with HH, but no formal training was provided to the subject ED staff.

### Measurement methods and main outcomes

Fifteen studies (75%) assessed primary outcomes according to the WHO-recommended 5-moments [34]. One paper assessed the microbial loads on the hands of the ED HCWs [28], one focused on the adherence rate to preventive measures (PM) against blood-borne disease. Martel et al. [34] centred on respiratory hygiene compliance, with HH as only one of its components. One article has as the primary endpoint the use of alcohol-based hand cleanser in the ED with non-sterile glove use, and Strauch et al. [41] emphasised decline in the rate of sick calls for nurses and technicians using an automated HHC system (HHCS). Almost all studies mentioned a certain degree of training of the involved observers, of which only three studies (15%) used qualified infection control experts [19, 37, 38]. Three studies (15%) used trained medical students as observers [25, 29, 34]. Seven studies (35%) used a single observer [15, 16, 32, 34, 37–39], three studies (15%) involved two observers [25, 27, 30]. Cartel et al. [26] included four observers, Martel et al. [34] worked with five medical students, and Venkatesh et al. [19] conducted their research with five trained observers and one qualified infection control specialist (Table 7). Coming to statistical analysis, fifteen studies (75%) used frequency percentage (%). Eight papers (40%) used the 95% Confidence interval (95% CI) in their studies. The P-value was described in twelve of twenty studies (60%). Mean and standard deviation for parametric data was used by five authors (25%) [15, 30, 33, 36, 40] while the median and interquartile range (IQR) was associated with four papers (20%) [28, 34, 35, 40]. Odd ratio was applied in four publications [15, 16, 34, 35]. Venkatesh et al. [19] brought in an adjusted risk ratio (aRR) (Table 8).

**Table 8:** Results

**Authors**	**Year**	**Statistical analysis**	**Number of opportunities**	**HHC at baseline and HHC post intervention**	**Conclusion**
Arntz et al. [25]	2016	-Frequency % -95% CI -P-value	1007 HHO	-18.2% (95% CI 14–22%) -After week 1: HHC rate 40.5% (95% CI 33–48%), *P* < 0.001 -After week 2: HHC rate 49.5% (95% CI 43–56%), *P* = 0.075 -After week 3: HHC rate 45.7% (95% CI 39–53%), *P* = 0.443 -HHC Nurses: 12.4% (95% CI, 8–14%) to 47.0% (95% CI, 30–48%; *P* < 0.001) -HHC physicians: 27.4% (95% CI, 20–34%) to 43.6% (95% CI, 26–45%; *P* = 0.013)	The multimodal HH strategy for nurses and physicians on the ED has substantial positive effects on HHC
Carter et al. [26]	2016	-Frequency % -OR -95% CI -P-value	1,673 HHO Nurses: 55% Physicians: 32% Nursing assistants: 10% Others: 3%	-HHC in non-crowding periods: 67% -HHC patients in private area: 74% -HHC moment 1: 23%, HHC moment 2: 7%, HHC moment 3: 19%, HHC moment 4: 39%, HHC moment 5: 12% -HHC night shift/day shift: OR = 1.37, 95% CI:1.04–1.80 -HHC physicians/nurses: OR = 1.60, 95% CI:1.25–2.04 -HHC ED overcrowded/not crowded: OR = 0.56, 95% CI: 0.42–0.75 -HHC ED severely overcrowded/not crowded: OR = 0.63, 95% CI: 0.46–0.86 -HHC ED dangerously overcrowded/not crowded: OR = 0.39, 95% CI: 0.28–0.55 -HHC in hallways/semi-private areas: OR = 0.73, 95% CI: 0.55–0.97	-Crowding was associated with lower hand hygiene compliance in the ED -Hallway care was associated with lower hand hygiene compliance
di Martino et al. [27]	2011	-Frequency % -P-value	-Preintervention: 420 (Doctors 181, Nurses 239) -Immediately post intervention: 463 (Doctors 200, Nurses 263) -1-year post intervention: 456 (Doctors 297, Nurses 159)	HHC Preintervention to immediately post intervention: -Overall: 14.3% 44.9% (*P* < 0.001) -Nurses: 19.2% 40.7% (*P* < 0.001); Absolute HHC 21.4% (*P* < 0.001) -Doctors: 7.7% 50.5% (*P* < 0.001); Absolute HHC 42.8% (*P* < 0.001) HHC 1-year post intervention: -Overall: 45.2%, -Nurses: 49.8%, -Doctors: 36.5% Change post intervention to 1 year (%): -Overall: 0.3% (*P* < 0.939), -Nurses: 9.1% (*P* < 0.30), -Doctors: -14.0% (*P* < 0.008)	The overall impact of intervention transpired over one year period, although a meaningful difference was evident between nurses and physicians
Espinoza Diaz et al. [28]	2018	-Median & IQR (25^th^-75^th^ percentile)		**Microbial load:** -Physicians: Median 90 CFU/g (40–180) -Interns: Median 335 CFU/g (60–785) -Residents: Median 82.5 CFU/g (50–160) -Nurses: Median 100 CFU/g (90–110) -Nurse assistants: Median 545 CFU/g (30–2300) **HH before & after procedures:** -Physicians: 40%, Interns: 70%, Residents: 60%, Nurses: 100%, Nurse assistants: 40% **Time for washing hand over 40 s:** Physicians: 60%, Interns: 60%, Residents: 60%, Nurses: Median 80%, Nurse assistants: 60%	The median CFU/g was identical to prior studies with auxiliary nursing staff recording the highest microbial load and the lower percentage of handwashing
Fouad et al. [29]	2020	-Frequency % -CFU/g -P-value	1374 HH opportunities **HHO Phase I:** -Nurses: 87.92% -Physicians: 10.73% -Other: 1.36% **HHO Phase III:** -Nurses: 79.49% -Physicians: 17.84% -Other: 2.67% **Overall HH0:** -Nurses: 83.55% -Physicians: 14.41% -Other: 2.04%	**WHO 5HH at baseline/ Phase I:** -HHC moment 1: 17.38%, HHC moment 2: 25.56%, HHC moment 3: 12.93%, HHC moment 4: 25.56%, HHC moment 5: 18.57% -Bacterial loads of hands: log CFU/hand 4.97 (0.32) **WHO 5HH at Phase III:** -HHC moment 1: 16.11%, HHC moment 2: 30.14%, HHC moment 3: 14.72%, HHC moment 4: 19.03%, HHC moment 5: 20.00% -Bacterial loads of hands: log CFU/hand 4.57 (0.47) (*P* < 0.001) -HHC increased in Physicians: 187% from baseline (*P* < 0.001) -HHC increased in Nurses: 147% from baseline (*P* < 0.001) -HHC increased in other: unchanged from baseline (*P* < 0.926)	HH educational programmes were effective in improving compliance in the ED and bacterial load on hands of HCWs could be used as an indicator of improvement in hand hygiene compliance
Ghazali et al. [30]	2018	-Mean & SD -Frequency % -95% CI -P-value	22 participants 218 HH procedures during (A) 220 HH procedures during (B)	**Assessment A (before SBT):** -Duration of HH: 31.2 ± 13.6 s -First HH procedure: 23.5 s (95% CI, 0.1–22.6) vs Tenth HH procedure: 22.7 s (95% CI, 1.2–30.4); (*P* = 0.07) -Number of steps: 6.03 ± 0.73 -HH score right palm (out of 18): 16.42 ± 0.46, -HH score left palm (out of 18): 16.12 ± 0.78, HH score right dorsum (out of 22): 15.46 ± 0.85, HH score left dorsum (out of 22): 16.0 ± 1.93, non-hygienic nails: 4.13% -Participants who took test: score 2.8 ± 0.2 **Assessment B (post SBT):** -Duration of HH: 35.8 ± 16.6 s (*P* = 0.04) -First HH procedure: 49.6 s (95% CI, 0.1–22.6) vs assessment A (*P* < 0.01) -First HH procedure: 49.6 s (95% CI, 0.1–22.6) vs tenth HH procedure: 27.4 s (95% CI, 2.9–4.9); (*P* = 0.01 -Duration of last HH B vs A: *P* = 0.03 -No difference between nurses, nurse assistants and residents: F = 0.372; *P* = 0.95 -Number of steps A vs B: 6.33 ± 0.92 (*P* = 0.13) -HH score right palm (out of 18): 16.50 ± 0.58; *P* = 0.15 -HH score left palm (out of 18): 16.95 ± 0.37; *P* = 0.02 -HH score right dorsum (out of 18): 17.0 ± 2.24; *P* < 0.001 -HH score left dorsum (out of 18): 18.05 ± 1.95; *P* < 0.001 -Non-hygienic nails: 0.0%; *P* = 0.02, -Presence of jewelleries A vs B: *P* = 0.85 -Presence of long nails A vs B: *P* = 0.08, -Presence of skin wound A vs B: *P* = 0.42 -Participants who took test: score 4.4 ± 0.1	An optimal number of steps were performed during hand-rubbing procedures and that SBT improved the duration and quality of hand hygiene, except for the dorsal right side. Emphasis should be placed on the specific hand areas that remained unclean after regular hand-rubbing procedures
Haac et al. [31]	2017	-Frequency % -Range	1034 HHO -30 patients -342 HCW-patient interactions	Patients in critical conditions: 47% Patients presented during the night: 57% Average HCW-patient interaction/patient: 11.4 (range 7–19) Average HH opportunity/patient: 3 (range 1–12) Average duration of HCW-patient interaction: 25 min HHC glove donning/removal doctors: 63% HHC glove donning/removal technicians: 62% HHC glove donning/removal students: 59% **WHO 5HH opportunities:** -Before patient contact: 36% (*n* = 375) -Before clean procedure: 17% (*n* = 178) -After contact with bodily fluid: 2% (*n* = 19) -After patient contact: 36% (*n* = 376) -After contact with patient surroundings: 8% (*n* = 86) **HHC using soap or water or alcohol gel:** -Before patient contact: 3% (10/375) -Before clean procedure: 0% (0/178) -After contact with bodily fluid: 11% (2/19) -After patient contact: 15% (57/376) -After contact with patient surroundings: 2% (2/86) -Gloves removed with no HH: 51% -New gloves donned before patient contact: 69% -New gloves donned before surgical procedures: 75% -Gloves removed after patient contact: 47% -Gloves removed after contact with bodily fluids: 58% -HHO Clean procedures: 17% of all HHO -Clean procedure prior device insertion: 42% (*N* = 76) -Clean procedure prior opening circuit or device: 32% (*N* = 52) -Clean procedure prior bedside surgical procedure: 11% (*N* = 20) -Clean procedure prior device insertion: 42% (*N* = 76) -HHC critical vs non-critical/ Day vs night shifts: *P* > 0.05 -HHC technicians:17% (*n* = 5/29) -HHC students: 13% (*n* = 5/39) -HHC glove donning/removal nurses: 51%	HH opportunities are frequent and compliance with WHO HH guidelines may be infeasible, requiring significant amounts of time that may be better spent with the patient during the golden hour of trauma resuscitation In an era where more scrutiny is being applied to patient safety, particularly the prevention of inpatient infections, more research is needed to identify alternative strategies (e.g., glove use, prioritizing moments) that may more effectively promote compliance in this setting
Hong et al. [32]	2012	-Frequency %	112 microbiological swabs: -56 keyboards -56 electronic mice Hospital A: 44 samples Hospital B: 42 samples Hospital C: 26 samples 2451 contacts with computer equipment (CE)	**Growth of organisms on culture**: 92% (103/112): -Multiple bacterial species: 33.9% (38/112) -Coagulase-negative streptococci: 85.7% (96/112) -MRSA: 1.8% (2/112 keyboards only) -No growth: 8% (9/112) -Keyboard contamination: 98.2% -Mice contamination: 85.7% -Keyboard contamination for multiple bacterial species: 50% (95% CI, 36%-64%) -Mice contamination for multiple bacterial species: 17.8% (95% CI, 9–30%) -HHC before contact with CE:29.9% -**Total Hand contacts and HHC:** HHC after patient contact: 38% (95% CI 35–40) vs HHC after other contact: 20.7% (95% CI 18–23); *P* < 0.001 -**Keyboards:** HC after patient contact: 42.2% (95% CI 38–47) vs HHC after other contact: 23.4% (95% CI 19–28); *P* < 0.001 -**Mice:** HHC after patient contact: 35.3% (95% CI 32–39) vs HHC after other contact: 19.2% (95% CI 16–22); *P* < 0.001	The rate of HHC before contact with computer equipment was very low in this emergency department setting (29.9%) Practices such as hand washing and barrier protection are considered the simplest and most important measures to prevent nosocomial infections in the hospital setting
Mahfoozpour et al. [33]	2013	-Mean & SD, -Frequencies % -P-value		-HH before gloving: Mean 0, SD 0, Poor 100%, Fair 0%, Good 0% -HH after Degloving: Mean 95, SD 20, Poor 5%, Fair 0%, Good 95% -Use of latex glove before procedures: Mean 38, SD 37, Poor 56.3%, Fair 11.2%, Good 32.5% -HH after Degloving per experience: 16.7% for 1^st^ year EMR, 27.8% for 2^nd^ year EMR and 46.8% for 3^rd^ year EMR (P = 0.003) -Respecting BDD instructions per experience: 19.3% for 1^st^ year EMR, 23.3% for 2^nd^ year EMR and 29.5% for 3^rd^ year EMR (*P* = 0.002) -HH after Degloving per procedure: 47% for IV-line preparation, 25% for intubation (*P* = 0.013) -Mean of respecting instructions to prevent BDD according to procedures: *P* = 0.065 -Respecting protocols during day vs night shifts EMR: *P* > 0.005	The preventive measures for BDD were not abided to as expected, this might be due to large number of patients and crowded wards, high workload and need for fast performances of duties. Training courses are necessary
Martel et al. [34]	2013	-Median & IQR, -OR -P-value	-118 patients with fever & cough out of 372 patient visits -115 observations (58 Hospital 1 and 57 hospital 2) -114 questionnaires distributed, 61% response rate	-Day shift observations: 61.7% -Evening shift observations: 33.9% -Night shift observations: 4.3% -Nurses: female 87%, male 13% -Overall respiratory compliance: 22% (IQR, 11–33%) -HH after patient contact: 53% -HH before patient contact: 43% -Overall actual knowledge median score: 75 (IQR, 75–100) -Physicians’ vs Nurses perceived knowledge score: 80 (IQR, 65–80) vs 60 (IQR, 60–80), *P* = 0.08	The compliance rate is very low. Identified factors affecting adherence to respiratory hygiene measures that are of potential use in targeting groups and formulating recommendations
Muller et al. [16]	2015	-Median & IQR, -OR -P-value	1116 HHO during 130 0bservations -Nurses: HHO 68% -Physicians = HHO 18% -Other = HHO 14%	-Median HHO per observation: 8 [IQR = 4–12] -Nurses: HHO 68%, HHC 30% -Physicians = HHO 18%, HHC 34% -Other = HHO 14%, HHC 19% -Mean HHC: 29% -ABHR HHC: 66% -Soap & water HHC: 33% -Median number patients per day: 195 [IQR = 184–208] -Median time from registration to physician assessment: 1.5 h [IQR = 1.2–1.7 h] -Median time from patient triage to discharge: 5.3 h [IQR = 4.8–5.5] -Median nursing hours per shift: 116 [IQR = 105–128] -Median number of nursing overtime hrs: 11.8 [IQR = 4–23]	
Parmeggiani et al. [35]	2010	-Frequency %, -OR, -95% CI, -P-value	550 surveys distributed 307 response rate (55.8%)	-HCWs wearing gloves, mask, and protective eyewear: 94.1% -HCWs HH after removing gloves: 91.5% -HCWs aware that their hands can transmit HAIs: OR = 4.64 (95% CI, 1.85–11.68); *P* = 0.001 -HCWs know the risk of transmitting HCV & HIV: OR = 6.07 (95% CI, 0.11–0.5); *P* = 0.021 -HCWs not know the risk of transmitting HCV & HIV: OR = 0.24 (95% CI, 1.31–28.14); *P* < 0.001 -HCWs using educational courses & scientific journals as source of information on HAIs: OR = 3.54 (95% CI, 1.47–8.5); *P* = 0.005 -HCWs know that HH after removing gloves is a HAIs control measure: OR = 8.09 (95% CI, 2.83–23.1); *P* < 0.001 -Nurses’ knowledge of protective measures (gloves, mask, eye protection) and HH: OR = 2.34 (95% CI, 1.09–5.01); *P* < 0.001	-HCWs have high levels of knowledge, positive attitudes, but low compliance concerning standard precautions -Nurses had higher knowledge, perceived risk, and appropriate HAIs control measures than physicians, and HCWs answered correctly and used appropriately control measures if have received information from educational courses and scientific journals
Reardon et al. [36]	2012	-Mean, 95% CI		-Total time for gloving and removal with hand rub: 44 s (95% CI, 39–49 s) -HH before gloving: 17 s (95% CI, 13–22 s): -HH: 11 s (95% CI, 9–13 s) -Donning gloves after HH: 6 s (95% CI, 3–10 s) -HH after removal of glove: 6 s (95% CI, 4–9 s) -Total time HH with non-sterile gloves: 24 s -Time Residents donned gloves: 0.8 times/hr (95% CI, 0.5–1.1) -Observed gloving episodes indicated: 77% (95% CI, 63–91%) -Number of new patient seen /resident/hr: 1.2 (95% CI, 1.0–1.5) -Number of times resident entered patient’s room or hall space: 3.7/hr (95% CI, 3.2–4.2) -HHC before glove donning: 44% (95% CI, 27–61%) -HH rub for 20 s or more: 3% (95% CI, 0–9% -HH with soap & water: 0%	ABHR use before and after non-sterile glove use represents a real but manageable additional time commitment, requiring 39–108 s per hour for a typical emergency physician
Sakihama et al. [37]	2015	-Frequency % -P-value	2982 observations	-Overall HHC preintervention: 18% -Overall HHC post intervention: 32.7% (*P* < 0.001) -Use of ABHR with appropriate HH: 90% in post intervention vs 67% in preintervention (*P* < 0.001) -Overall HHC in the ED: -ED Nurses and physicians showed significant decrease in adherence rate 15,7% at baseline to 7.4% post intervention (*P* = 0.02)	Using a novel contest coupled with a multimodal intervention successfully improved HHC rates. However, further improvement is necessary
Scheithauer et al. [38]	2013	-Frequency % -P-value	5674 HHO 1664 HR during tri-phase study period	-HR increases from phase I (21%) to III (45%), *P* < 0.001 -Trainees: 61 HR, 205 HHO, 30% HHC phase I, 33% HHC phase III -Nurses: 695 HR, 2448 HHO, 18% HHC phase I, 45% HHC phase III -Physicians: 600 HR, 1889 HHO, 26% HHC phase I, 43% HHC phase III -HH Moment 2: 660% from baseline (Highest) -HH Moment 4:150% from baseline (Lowest) -Neurological patients: HR347, HHO 1378 -Medico-thoracic patients: HR79, HHO 1307 -Surgical patients: HR411, HHO 1442	
Schmitz et al. [39]	2014	-Frequency % -95% CI -P-value	2000 HHO (Baseline 1000 HHO and Post-intervention 1000 HHO)	-Number of sinks with Hand washing materials: 20% -Entire hospital HHC at baseline: 2.1% -ED HCWs HHC: 5.1% -Entire hospital HHC post-intervention: 12.7% (OR = 6.8, 95% CI 4.2–10.9) -ED HCWs HHC: 24.8% (*P* < 0.001) -Univariate analysis of predictor of HHC in ED: OR 5.1 (95% CI, 3.0–8.5); *P* < 0.001 -Multivariate analysis of predictor of HHC in ED: OR 4.9 (95% CI, 2.8–8.6); *P* < 0.001 -ED HCWs completed postintervention survey: 6.2%	There was a significant increase in HHC among Ethiopian HCWs following the implementation of WHO-recommended multimodal HH campaign (WHO 5HH)
Stackelroth et al. [40]	2015	-Mean & SD -Median & Range	459 HH episodes	-ED staff: 92.4%, -Non-ED staff 1.3%, -Unable to determine 6.3% -Soap-based wash: 98.1% -Alcohol based foam: 1.9% -Product used correctly: 93.8% -Product used incorrectly: 6.2% -Did not hesitate, immediately selected HH product: 91.8% -Moved to use one product but stopped and used another: 3.3% -Applied one product then changed to another: 2.4%	The amelioration of causes of error and hesitation by standardization of the appearance and relative positioning of HH solutions at washbasins may translate into improved HH behaviours. Placement of moisturizer at the washbasin may not be essential
Strauch et al. [41]	2020	-Frequency % -95% CI		-Preintervention average monthly HH: 94% -Reduction of monthly sick call hours after HHCS: 4.6% (95% CI, 0.6–8.7%); *P* = 0.32 -Decreased overtime hours worked by substitute staff: 11,949 (2015) to 7,092 (2018) -Decreased overtime hours worked per employee: 31 (2015) to 17 (2018)	A reduction in employee absenteeism was an unexpected result of implementation of an automated HHCS
Venkatesh et al. [19]	2011	-Frequency %, -Adjusted RR	5865 HHO	-Overall HHC: 89.7% (95% CI, 88.9–90.5%) -Physician assistant HHC: 96.7%, -Physician HHC: 91.9%, aRR = 0.97 (95% CI, 0.95–0.99) -Nurse HHC: 89.4%, aRR = 0.94 (95% CI, 0.93–0.96) -Nurses assistant HHC: 89.6%, aRR = 0.96 (95% CI, 0.93–0.98) -Other HHC: 86.8%, aRR = 0.95 (95% CI, 0.92–0.99) -Transport HHC: 63.3%, aRR = 0.67 (95% CI, 0.60–0.75) -General ED HHC: 90.4% -Observation unit HHC: 84%; aRR = 0.93 (95% CI, 0.86–0.96) -Private bed HHC: 90.8% -Hallway HHC: 82.3%; aRR = 0.89 (95% CI, 0.86–0.92) -No Glove use HHC: 91.5% -Glove use HHC: 83.3%; aRR = 0.98 (95% CI, 0.89–0.94) -HHC before patient contact: 90.3% -HHC after patient contact: 89.5% -HHC on weekend: 91.4% -HHC on Weekday: 89.7% -HHC on Day shift: 90.0% -HHC on night shift: 89.6%	HHC in the ED is associated with both traditional and ED-specific characteristics. Patients receiving care in hallway location were less likely to have caregivers properly perform HH
Zottele et al. [15]	2017	-N, % of frequencies for qualitative variables -Means & SD: for quantitative variables -OR & 95% CI to compare proportions	166 HHO during 111 days of monitoring	Mean time interval between 2 sessions: 28 days (SD = 9 days) Time lasted for observation session: 11 min (SD = 50 s) **Morning shift/Session 1:** -Observation: 46% -HHO: 35.5% -Mean duration: 10 min 20 s (SD = 4 min 52 s) -HHO with soap: 77% -HHO with ABHR: 23% **Afternoon shift/Session 2:** -Observation: 20% -HHO: 33% -Mean duration: 11 min 50 s (SD = 5 min 21 s) -HHO with soap: 83% -HHO with ABHR: 17% **Night shift/Session 3:** -Observation: 34% -HHO: 31%, -Mean duration: 10 min 40 s (SD = 4 min 38 s) -HHO with soap: 87% -HHO with ABHR: 13% Overall HHC: 54.2% HHC Session 1: 50.8% HHC Session 2: 52.7% HHC Session 3: 59.6% HHC nurses: 66.6% HHC nurse’s technicians: 50.6% HHC physiotherapists: 66.6% HHC resident physicians: 41.3% HHC nurse’s vs resident physicians: OR = 2.83; 95% CI, 1.09–7.34), *P* = 0.03	The HHC rate in adult Ed was low at 54.2%. Multidisciplinary approaches could be important strategies for forming partnerships to develop learning and implementation of HH practices

### Hand hygiene opportunities

Fourteen studies described 25,192 HHOs and 1664 hand rubs (HR) across all HCWs observations. The highest opportunities were described by Scheithauer et al. [38] and Venkatesh et al. [19], respectively, with 7338 (5674 HHOs and 1664 HR) and 5865 HHOs. Zottele et al. [15] reported the lowest number with 116 HHOs. Other studies recorded considerably less participants and HHOs; specifically: Ghazali et al. [30] 22 participants, < 220 HHOs; di Martino et al. [27] conducted 3 intervention phases referring to > 420 HHOs in each; Cartel et al. [26] appraised nurse HHO rate (55%) comparing to that of physicians (32%) or Nursing assistants (10%) (Table 7); Fouad et al. [29] reported similar HHOs among nurses (83.55%) and physicians (14.41%); as did Scheithauer et al. [38]. Muller et al. [16] found that nurses had 68% HHO, physicians totalling 18%, and others (nursing assistant, housekeeping, transport, IV team, radiology technicians, and nursing students) scoring 10% (Table 8).

Distinctly, Espinoza Diaz et al. [28] conducted a microbiological evaluation of HHC stating high HH compliance amongst nursing staff, higher than interns, residents, physicians, and nurse's assistants respectively. Hong et al. [32] swabbed ED electronic devices (56 keyboards and 56 mice), while Martel et al. [34] targeted respiratory compliance among ED HCWs. Similarly, Parmeggiani et al. [35] surveyed ED staff with a 55.8% response rate out of the 550 questionnaires distributed. Reardon et al. [36] evaluated the use of ABHR and Mahfoozpour et al. [33] assessed preventive measures against blood-borne disease, but without providing details of HHO observed.

### Hand hygiene compliance

Overall, six studies [25, 27, 35, 37–39]  documented baseline HHC rates, with an overall median of 14% (range 5.1–21%). Typically, nurses displayed higher averages (median 17%, range 3.5–31.4%) than physicians (median 11.5%, range 2.9–26%). Of note, post-intervention, all studies reported an improvement in HHC rate to an overall median of 45%, ranging between 8% and 89.7% (Table 9 in the Appendix); again nurses improved HHC most (median 46%, range 8–100%) than physicians (median 40%, range 5.8–91.9%). Of these studies, three adopted multimodal approaches successfully [25, 37, 39]. However, sustainability of improvements is questioned by di Martino et al. [27], who appraised compliance in the ED a year following intervention, and noted disempowerment from initial high levels achieved.

From a microbiology perspective, it was notable that some groups investigated microbial burden on hands as surrogate markers of HHC [28, 29]. Specifically, Espinoza et al. [28] found higher bacterial loads on nursing assistants’ hands (545 CFU/g, IQR 30–2300) than on interns’ hands (nurses or medical) (335 CFU/g, IQR 60–785), while nurses, physicians, and residents yielded significantly lower levels (Table 8). Alternative approaches assessed computer accessories, with Hong et al. [32] reporting contamination of 92% of equipment culture with significant levels of culturable species, including potentially pathogenic isolates from 50% of samples.

### Impact of overcrowding and workload

Overcrowding and high numbers of transiting patients are features of EDs. In that context, studies focused on influence of overcrowding on HHC reported direct relationship between overcrowding and poor HHC [16, 19, 26[. Of these, Cartel et al. [26] observed poor HHC elevated by 63% during overcrowding (OR = 0.63, 95% CI: 0.46–0.86) (Table 8), while Muller et al. [16] ascertained a direct correlation between overcrowding and poor soap and water HHC (33%) or ABHR (66%). (Mean = 29%).

Mahfoozpour et al. [33], when assessing the rate of adherence to preventive measures (PM) amongst 80 EM residents (EMR), determined poor behaviour as being related to workload and the need for speed when performing ED clinical duties [33] (Table 8). Interestingly then, Ghazali et al. [30] employed simulation-based training (SBT) to estimate the duration and the quality of HH before and after the SBT, reporting an increase of the duration of HH from 31.2 s (± 13.6 s) at baseline to 35.8 s (± 16.6 s, P = 0.04) post-SBT, probably reflecting rushed practices in real-world scenarios settings. Indeed, Reardon et al. [36] quantified the time burden of ABHR linked with the use of non-sterile glove by ED HCWs, determining that only 3% (95% CI, 0–9%) of the participants adhered to WHO recommendations of 20 s of hand rubbing.

### Evaluating with technological aids

Assessment of HHC using technological tools Is not new but remains uncommon. In the ED setting, Haac et al. [31] used video surveillance in resuscitation bays to appraise HHC, while Stackelroth et al. [40] used 24 h-period video surveillance of ED HCWs staff and non-ED staff (EMS personnel) (Table 8). Perhaps most usefully, Strauch et al. [41] introduced electronic badges to remind staff of maintaining high standards of HHC. Participants achieved a 94% HHC rate.

### WHO 5 moments

Despite ubiquitous information regarding the WHO 5 moments, Zottele et al. [15] analysed ED HHC based on the WHO 5-moments of HH and noticed a profoundly low rate (54.2%), with nurses demonstrating a higher compliance rate than physicians (66.6% vs 41.3%, OR = 2.83, 95% CI, 1.09–7.34). Other studies [16, 25, 29–31] detailed HHC rates for the distinct moments, with moment-1 (before touching a patient) ranging between 3% [31] and 36% [30], moment-2 (before clean/aseptic procedures) between 0% [31] and 25.5% [29], moment-3 (after body fluid exposure) between 2% [30] and 26% [16], moment-4 (after touching a patient) between 15% [31] and 31.6% [25] and moment-5 between 2% and 18.5% [29].

## Discussion

While there have been previous reviews of hand hygiene practices, and associated interventions for improvement, across healthcare settings generally, there has been comparatively little emphasis placed on emergency facilities specifically. In that context, this review focuses on emergency departments, with particular discussion of the hand hygiene challenges that may be encountered due to large numbers of patients transiting services, often requiring urgent care that is frequently delivered in overcrowded spaces. This review provides a comprehensive systematic appraisal of published studies of hand hygiene and interventions relevant to this setting in particular.

### Hand hygiene compliance

Twelve of the twenty included studies reported participant HHC. In the nine observational studies in which HHC was documented, we calculated a median post-intervention HHC rate of 45% (range 8–89.7%) (Table 9 in the Appendix). This rate correlated with those detailed by Cartel et al. (7.7 to 89.7% in a review of studies published between 2002 and 2012) [2] and Seo et al. (7 to 89% in 12 cross-sectional studies identified in a search of literature published between 1948 and 2018) [19]. Encouragingly, we determined a slightly higher HHC rate than 40% HHC described by Erasmus et al. [43] in 2010. It is, perhaps, reasonable to suggest that this modest increase in HHC rate can be attributed to awareness of the WHO recommended 5-moments of hand hygiene in the last decade promoted through education and training campaigns.

Critical appraisal of the observational studies eligible for our review highlighted complexity in both data extraction and comparison across publications. Specifically, while eight of the nine studies defined HHC according to the WHO recommended 5-moments, Muller et al. [16] combined moments 4 and 5 as a single moment. Further variation in methodology related to choice of time points when HHC was to be determined. For instance, some studies observed HHC immediately after intervention [16, 25, 26, 29, 37], while others delayed observation from six weeks [27, 38] to a year post-intervention [27]. There was similar diversity across with regard to whether HHC was monitored before and after a patient contact [33, 34] or before and after gloving [36] or before and after entering the patient room [41]. However, the greatest disparities related to HH techniques employed, participant educational attainment, cohort sample sizes, and ED level of activity during period of observation (especially staffing level and workload, and overcrowding). To exemplify the impact of such variation, in an African study published in 2014, Schmitz et al. [39] described a post-interventional average HHC rate of 24.8%, representing a HHC rate of almost twice the 14.9% average detailed five years later by Engdaw et al. [44] in the same country. However, the two studies are not comparable directly as, unlike the participant cohort studied by Schmitz et al. based in academic institutions in Addis Ababa [39], that of Engdaw et al. worked at a district level in primary health care centres [44].

Despite a paucity of analogous studies, there is an evident association of ED overcrowding with poor HHC [15, 16, 26].

### Hand hygiene interventions

Like others, we observed the most effective interventions ED HCWs to be education and training either alone or complemented by posters, feedbacks, presentations, live demonstration, simulation, or video surveillance. Exemplifying this, Ward et al. [45] found that interventions such as reminder sounds, practical simulations, videos, and audio-visual media significantly improved handwashing compliance. Seo et al. [20] affirmed that the use of multimodal tactics was an effective way to promote HHC among ED HCWs, while Sakihama et al. [37] commented favourably on addition of contests successfully improved HHC. The latter approach was similarly highlighted by Luangasanatip et al. [46] in their study of comparative efficacity of HH interventions where they introduced reward incentives and accountability metrics.

Irrespective of the potential of such approaches, ED HHC rates remain relatively poor. Interventions may be hampered by ED overcrowding [16, 26] or the amount of time it takes to follow the WHO steps while dealing with a real emergency that may be perceived as barriers. It appears that further robust studies, preferably randomized controlled trials or interrupted time series, are required to draw definitive conclusions as to which interventions may be appropriate and effective in influencing HHC in ED settings.

### ED healthcare workers

Across the twenty studies eligible for our review, 19 involved ED nurses, with 16 studies focused on physicians (80%). Of note, nurses demonstrated higher HHC compared to physicians and other personnel with an overall mean rate of approximately 46% (8–100%), exceeding that of physicians at around 40%. Proportionally, this is similar to the findings of Erasmus et al. [43] who observed physician HHC to be lower (32%) than nurses (48%).

These observations, however, are challenging as, unfortunately, the definition of physician and nurse have not been consistent across studies. For example, the term “physicians” may represent residents, consultants, general Doctors, or trainees; yet the denomination "physician" was applied in multiple studies without clarity regarding actual clinical grade. Five studies [25, 27, 30, 33, 36] provided data relating to differentiated ED HCWs, while two studies presented data attributable to all ED HCWs collectively [35, 39]. It seems reasonable to suggest that studies ought to refine their design to better understand the impact of experience and clinical responsibility across physician and nursing staff. Similar attention to detail should be applied to investigation of all other staff, clinical, allied health professionals or support staff (porters, hygiene or catering staff) and their contribution to ED HHC and HCAI rates.

### Geographical region

Employing World Bank region and income classification [24], we found that of the twenty eligible papers sixteen studies (80%) were conducted in higher-income countries (Europe, North America, East Asia and Pacific and Middle East Asia), while one originated from Ethiopia [39] and three (15%) from upper-middle-income countries (Latin America and the Caribbean and Middle East Asia) [15, 27, 33].

While Africa is home to 17% of the world's population [47], Sub-Saharan EDs reported only one study. Latin America and the Caribbean were represented twice (10%). Asia, with close to 60% of the world population, contributed 25% of the papers. Europe, with 9%, and North America, with 5% of the world population [47] provided 60% of eligible studies. This aligns with a large review conducted by Clancy et al. [3] who commented that 79% of HH studies originated from Asia, Europe, and North America combined.

These evident disparities are, of course, influenced considerably by availability of resources to either perform studies or publish them. However, taken at face value, there are many determinants that may explain the poor HH practices in low- or middle-income countries. Engdaw et al. [44] stated that half of participants had no access to sinks and ABHR, therefore a major factor in poor HHC. Other influencers include lack of basic infrastructure and equipment; limited financial support; inadequate healthcare systems; scarcity of satisfactory HH training; deficiencies in infection control and prevention programs; and poorly developed hand hygiene awareness and attitudes [44].

### Training and level of knowledge of ED HCWs

It is noteworthy that a small number of studies attempted to quantify the impact of knowledge and training on ED HHWs’ HHC. Eighteen of the studies referred to training of the involved observers. Amongst them, Parmeggiani et al. [35] utilised a self-reported survey to understand the level of knowledge by ED HCWs and determined that those who use gloves when at direct contact with a patient are 8 times more aware that HH following glove removal is a HCAI control measure; displaying higher compliance rates. In Kuwait, Al-Wazzan et al. [48] concluded that those with a good understanding of HH demonstrated almost seven times more adherence than those with lesser knowledge. Clearly, however, self-reporting is less than accurate and ethical studies utilising anonymous video surveillance are probably more accurate and reflective of actual practice [31, 39, 40, 45, 49], albeit those considerable resources are required to perform such research.

In conclusion, our results indicate that studies conducted in ED on HHC are essentially observational, weak, and prone to multiple biases. Hence, outcomes lack external validity and are difficult to be generalise. ED overcrowding and associated stressors are major barriers to HCW HHC in the ED. An unpredictable environment may require an adjusted strategy to improve HHC rates in the context of critically ill or polytrauma patients. A prudent recommendation may be to simplify the WHO advice and to emphasise the value of ABHR with gloves in improving HHC. Stating the obvious, there is an urgent need for robust and well-designed research to better approach ED poor HHC rates.

## Appendix

**Table 9:** Compliance at baseline vs post-intervention

Authors	Year	HHO Baseline (%)			HHC baseline (%)			HHC post-intervention (%)		
		Overall	Nurses	Physicians	Overall	Nurses	Physicians	Overall	Nurses	Physicians
Arntz et al. [25]	2016				18.2			45.5*		
Carter et al. [26]	2016		55	32					52.5	59.3
di Martino et al. [27]	2011				14.3	19.2	7.7	45.2	49.8	36.5
Espinoza Diaz et al. [28]	2018								100	40
Fouad et al. [29]	2020					31.44	23.94		46.11	44.88
Ghazali et al. [30]	2018									
Haac et al. [31]	2017									
Hong et al. [32]	2012									
Mahfoozpour et al. [33]	2013									
Martel et al. [34]	2013									
Muller et al. [16]	2015		68	18				29	30	34
Parmeggiani et al. [35]	2010				11	13	9	8	8	12
Reardon et al. [36]	2012									
Sakihama et al. [37]	2015				15	16	14	16	27	16
Scheithauer et al. [38]	2013		43	33	21	18	26	45	45	43
Schmitz et al. [39]	2014				5.1	3.5	2.9	24.8	19.3	5.8
Stackelroth et al. [40]	2015									
Strauch et al. [41]	2020									
Venkatesh et al. [19]	2011							89.7	89.4	91.9
Zottele et al. [15]	2017							54.2	66.6	41.3
Total			166	83	84.6	101.14	83.54	357.4	533.71	424.68
Mean			55.33	27.66	14.1	16.85	13.92	39.71	48.51	38.60
Median			68	32	14.65	17	11.5	45	46.11	40
Range			43–55	18–33	5.1—21	3.5–31.44	2.9–26	8–89.7	8–100	5.8–91.9

*This value is the average(mean) of the 3 interventions periods (40.5%, 49.5% and 45.7%)

## Abbreviations

5HH: 5-moments for hand hygiene: (1) before touching the patient, (2) before clean/aseptic procedure, (3) after body fluid exposure risk, (4) after touching the patient, (5) after touching patient surroundings
ABHR: Alcohol-based hand rub
ARR: adjusted Risk Ratio
BDD: blood–borne disease
C-DIFF: *Clostridium difficile*
CE: Computer equipment (s)
EMR: Emergency medicine residents
HAIs: Hospital Acquired Infection (s)
HCV: Hepatitis C Virus
HH: Hand hygiene
HHC: Hand hygiene compliance
HCW (s): Health care worker (s)
HHCS: Hand hygiene compliance system
HHO: Hand hygiene opportunity
HIV: Human Immuno-deficiency Virus
HR: Hand rub
HW: Handwashing
IQR: Interquartile range
PM: Preventive measures
SOPS: Standard operating procedures
TRU: Trauma Resuscitation Unit,
WHO 5HH: World Health Organisation recommended 5-moments for hand hygiene
